# *In vivo* client proteins of the chaperonin GroEL-GroES provide insight into the role of chaperones in protein evolution

**DOI:** 10.3389/fmolb.2023.1091677

**Published:** 2023-02-10

**Authors:** Hideki Taguchi, Ayumi Koike-Takeshita

**Affiliations:** ^1^ Cell Biology Center, Tokyo Institute of Technology, Yokohama, Japan; ^2^ Department of Applied Bioscience, Kanagawa Institute of Technology, Atsugi, Kanagawa, Japan

**Keywords:** chaperonin, GroEL and GroES, protein aggregation, chaperone, chaperone clients

## Abstract

Protein folding is often hampered by intermolecular protein aggregation, which can be prevented by a variety of chaperones in the cell. Bacterial chaperonin GroEL is a ring-shaped chaperone that forms complexes with its cochaperonin GroES, creating central cavities to accommodate client proteins (also referred as substrate proteins) for folding. GroEL and GroES (GroE) are the only indispensable chaperones for bacterial viability, except for some species of Mollicutes such as *Ureaplasma*. To understand the role of chaperonins in the cell, one important goal of GroEL research is to identify a group of obligate GroEL/GroES clients. Recent advances revealed hundreds of *in vivo* GroE interactors and obligate chaperonin-dependent clients. This review summarizes the progress on the *in vivo* GroE client repertoire and its features, mainly for *Escherichia coli* GroE. Finally, we discuss the implications of the GroE clients for the chaperone-mediated buffering of protein folding and their influences on protein evolution.

## 1 Introduction

Protein functions depend on their tertiary structures, which are dictated by their amino acid sequences, as demonstrated by Christian Anfinsen more than half a century ago (Anfinsen, 1973). Although protein folding is a spontaneous process in principle, folding frequently competes with the side process of aggregate formation, which is repressed by a variety of molecular chaperones (Dobson, 2003; Richter et al., 2010; Balchin et al., 2016). Indeed, a proteome-wide aggregation analysis of thousands of *Escherichia coli* proteins, using a reconstituted cell-free translation system, found that around 30% of proteins tend to aggregate without chaperones (Niwa et al., 2009), and the majority are saved by conserved chaperones such as the chaperonin GroEL/GroES (GroE) or DnaK/DnaJ/GrpE (DnaK system) (Niwa et al., 2012).

Chaperonins, a subclass of conserved chaperones, are responsible for promoting protein folding in cells (Thirumalai and Lorimer, 2001; Taguchi, 2005, 2015; Hayer-Hartl et al., 2016; Weiss et al., 2016; Thirumalai et al., 2020; Horovitz et al., 2022). The best-characterized chaperonin is *E. coli* GroE. GroE is a heat shock protein and the only indispensable chaperone for bacterial viability (Fayet et al., 1989), except for some species of Mollicutes such as *Ureaplasma* and *Mycoplasma* (Glass et al., 2000; Clark and Tillier, 2010; Schwarz et al., 2018). GroE helps fold numerous client proteins within cells using ATP. ATP binding to the GroEL rings induces drastic conformational changes leading to the formation of GroEL-GroES complexes, which have central cavities for client protein encapsulation (Xu et al., 1997; Kanno et al., 2009; Chen et al., 2013). The encapsulation of client proteins in the chaperonin cavity is essential to the growth of *E. coli* (Koike-Takeshita et al., 2006). An *in vitro* analysis revealed that the GroE cavity could accommodate proteins up to ∼60 kDa (Sakikawa et al., 1999). Although this review does not cover the detailed molecular mechanism of GroE, the basic role of GroEL is to bind to non-native monomeric proteins that arise after translation or during heat stress to prevent aggregation, and then promote folding in an ATP- and GroES-dependent manner.

In this review, we summarize the progress on the *in vivo* client proteins of the chaperonin GroEL and GroES (*in vivo* GroE clients) and discuss the roles of chaperonins in the cell. Other details on chaperonins have been summarized in recent excellent reviews (Weiss et al., 2016; Balchin et al., 2020; Horovitz et al., 2022).

## 2 Premise of the chaperone requirement: Proteins are aggregation-prone

**FIGURE 1:**
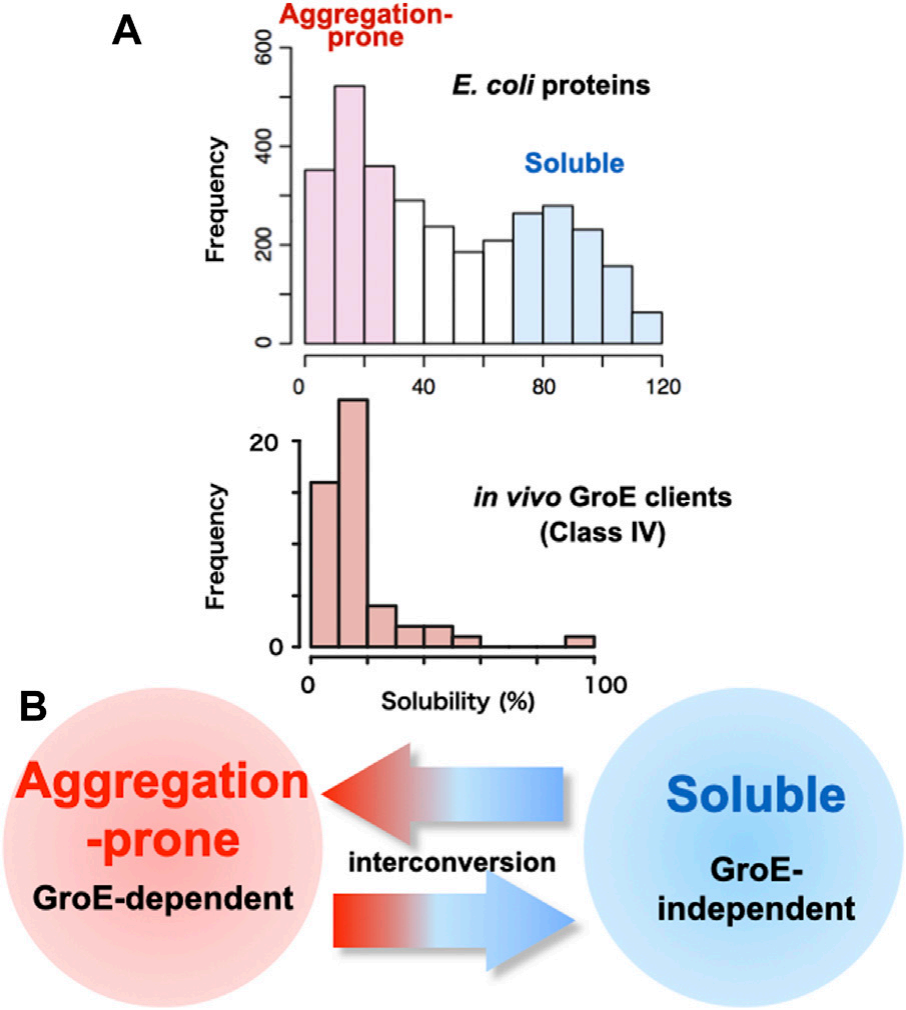
Relationship between GroE-dependency and protein solubility **(A)** Histograms of solubility distributions for 3,173 *E. coli* proteins (Niwa et al., 2009) and obligate GroE-dependent clients (Class IV clients) (Fujiwara et al., 2010). Inherent protein solubilities under chaperone-free conditions were determined by a global aggregation analysis, using a reconstituted cell-free translation system (PURE system). Solubility scores, representing the index of the aggregation propensity, are defined as the proportion of the supernatant fraction, obtained after the centrifugation of the translation mixture, to the uncentrifuged total protein (Niwa et al., 2009). **(B)** Interconversion of the aggregation-prone propensity and the GroE-dependency.

One of the reasons why chaperones are necessary for the cell is that proteins often aggregate. Indeed, researchers handling proteins know empirically that some proteins tend to form aggregates. What fraction of proteins is aggregation-prone at the proteome level? In this regard, a comprehensive study was conducted in which more than 70% of *E. coli* proteins, 3,173 proteins, were translated under chaperone-free conditions to determine whether they formed aggregates or were soluble (Niwa et al., 2009). The large-scale analysis using a reconstituted cell-free *E. coli* translation system (PURE system) revealed that the distribution of solubilities was bimodal, indicating that the *E. coli* proteome is divided into two groups: Soluble and aggregation-prone (Figure 1A). The analysis also showed that one-third of the proteins were aggregated when translated in the absence of chaperones, supporting the empirical view that proteins are aggregation-prone. A bioinformatics analysis demonstrated that protein solubility correlates better with cellular abundance, rather than gene-expression levels (Castillo et al., 2011). Subsequent analyses with the DnaK system or GroE showed that the addition of the chaperones during the translation of those aggregation-prone proteins alleviated aggregation overall, indicating the necessity of chaperones at the proteome level (Niwa et al., 2012).

Aggregation formation is associated with impaired folding. Recent global refolding experiments using an *E. coli* lysate, combined with mass spectrometry-based proteomics, revealed that one-third of the *E. coli* proteome is not intrinsically refoldable (To et al., 2021).

As these large-scale studies suggest, a certain fraction of proteins does not fold easily and often aggregates, supporting the notion that chaperones are essential to maintain the protein homeostasis (proteostasis) in the cell.

## 3 *In vivo* clients of GroEL-GroES

In *E. coli*, three major chaperone systems, trigger factor (TF), the DnaK system, and the chaperonin GroE, are considered to contribute to the folding of newly synthesized polypeptides. These three chaperone systems work together in a cooperative manner, with TF and DnaK both playing similar roles during co-translational processes *in vivo* (Deuerling et al., 1999; Teter et al., 1999; Ferbitz et al., 2004), whereas it is thought that GroEL plays a role in folding polypeptides after they have left the ribosome, although there have also been reports of GroEL potentially being involved in the co-translational process (Genevaux et al., 2004; Vorderwülbecke et al., 2004; Ying et al., 2005, 2006). An important goal in understanding the role of GroE in the cell is to identify *in vivo* obligate GroE clients that absolutely require GroE for folding in cells. The determination of the obligate GroE clients should clarify GroE’s unique role among chaperones, provide insight into the structural characteristics of the obligate clients, and illuminate the role of GroE in protein evolution.

### 3.1 Phenotype analyses using GroE-knockdown strains

Since the GroE-deletion *E. coli* strain is not available, due to the fact that GroE is the only indispensable chaperone for *E. coli* viability (Fayet et al., 1989; Horwich et al., 1993), a conditional GroE expression strain has been used to identify the *in vivo* GroE clients by analyzing the phenotype after GroE-depletion (McLennan and Masters, 1998). The depletion of GroE in *E. coli* has led to the identification of DapA and FtsE as essential clients in the cell lysis and filamentous morphology phenotypes, respectively (McLennan and Masters, 1998; Fujiwara and Taguchi, 2007). Although a detailed phenotypic analysis can precisely identify obligate GroE clients, this approach is limited because it can only identify one client at a time and only in cells with experimentally tractable phenotypes.

GroE depletion in *E. coli* causes the aggregation or degradation of newly translated polypeptides due to misfolding (*e.g*., Chapman et al., 2006; Fujiwara et al., 2010; Niwa et al., 2022). The use of mass spectrometry (MS) has identified around 300 proteins in aggregated proteins in a severe temperature-sensitive GroE strain, which harbors the GroEL (E461 K) mutant instead of wild-type GroEL (Chapman et al., 2006).

### 3.2 *In vivo* GroEL interactors

Another method to identify *in vivo* GroE clients is through a proteome-wide analysis of GroE complexes, including client proteins. Hundreds of GroEL interactors have been identified using MS (e.g., Houry et al., 1999; Kerner et al., 2005). In particular, Kerner et al. identified ∼250 *E. coli* proteins that are encapsulated in a chaperonin complex between *E. coli* GroEL and *Methanosarcina mazei* GroES, which tightly binds *E. coli* GroEL (Kerner et al., 2005). The interactors were categorized into three classes depending on their enrichment in the GroEL-GroES complex: Class I clients as spontaneous folders, Class II as partial GroEL-dependent clients, and Class III as potential obligate GroE clients (Kerner et al., 2005).

Note that other approaches to identify *in vivo* GroEL interactors have been applied to other bacteria besides *E. coli*. In *Thermus thermophilus*, MS-based proteomics of the endogenous *T. thermophilus* GroEL-GroES complex identified 24 clients in the chaperonin cavity (Shimamura et al., 2004). In *Bacillus subtilis*, a single-ring GroEL variant with a histidine-tag was used to identify 28 GroEL interactors (Endo and Kurusu, 2007). The archaeon *M. mazei* has the unusual property of possessing a group I chaperonin (GroEL) in addition to a group II chaperonin (Figueiredo et al., 2004). In this archaeon, *Methanosarcina* GroEL interactors have been identified by a large-scale co-immunoprecipitation analysis (Hirtreiter et al., 2009).

### 3.3 *In vivo* obligate GroE-dependent clients

An analysis of *E. coli* chaperonin GroEL interactors at the proteome level identified a group of proteins referred to as Class III clients, which were thought to be obligate chaperonin clients. However, the necessity of chaperonins for *in vivo* folding has not been thoroughly examined. In fact, one of the Class III proteins, ParC, was functional even under GroE-depleted conditions (Fujiwara and Taguchi, 2007), raising the possibility that the predicted Class III proteins are not necessarily obligate clients of GroE. A systematic assessment of the GroE requirement under the GroE-depleted condition revealed that ∼60% of the Class III clients required GroE for proper folding, and thus were regarded as *bona fide* obligate GroE clients *in vivo* and reclassified as Class IV clients (Fujiwara et al., 2010). Besides the Class III clients, a metabolomics analysis and the Class IV homologs in *E. coli* were used to identify additional Class IV clients (Fujiwara et al., 2010). In the metabolomics analysis, if the level of a metabolite is altered in a GroE-deficient strain, then the enzymes involved with the metabolite may be GroEL clients (Fujiwara et al., 2010). Furthermore, with the aid of data from the *in vitro* comprehensive analysis using the PURE system, 20 additional Class IV clients were identified (Niwa et al., 2016). In total, about 80 proteins have now been identified as Class IV clients.

It is worthwhile to compare these Class IV clients with other client candidate lists identified by various approaches or in other bacteria. Regarding the ∼300 proteins aggregated in the GroEL (E461 K) mutant strain, identified by Chapman *et al.* (Chapman et al., 2006), only 17 proteins were overlapped with Class IV clients (Fujiwara et al., 2010). The poor overlapping of the clients in these studies would be due to the different approaches used. Indeed, Masters *et al.* observed that insoluble proteins did not accumulate in GroE-depleted MGM100 cells (Masters et al., 2009), suggesting that the cellular milieus in the GroEL mutant strain and the GroE-depleted cells are quite different. Comparisons of Class IV clients in *E. coli* with GroE interactors in *T. thermophilus* and *B. subtilis* (Shimamura et al., 2004; Endo and Kurusu, 2007) revealed no apparent overlap, probably due to the fact that MS-based proteomics was in its initial stages in the early 2000s and the number of identified proteins was extremely small.

Collectively, the term “client” can vary significantly depending on the context in which it is utilized. The most commonly employed method for identifying chaperone client candidates is through the utilization of *in vivo* interactors obtained *via* pull-down assays. However, in the case of GroE, additional validation is necessary as GroE-interactors such as Class III proteins do not necessarily require GroE for *in vivo* folding. To qualify as a *bona fide* GroE client, folding in a GroE-depleted strain should be examined. Furthermore, *in vitro* experiments could provide greater insight into whether the folding of candidate clients can be aided by GroE during translation or after denaturation. In reality, however, determining whether a protein has completed folding correctly *in vitro* can be challenging. For enzymes, it is possible to assess folding completion through enzymatic activity, however, this activity-based approach is not universally applicable to all potential clients.

### 3.4 Features of the *in vivo* obligate GroE clients

The identification of the Class IV clients revealed several of their features, as follows (Fujiwara et al., 2010).

#### 3.4.1 Aggregation-prone properties

The most striking feature of the Class IV clients is their inherent highly aggregation-prone nature (Fujiwara et al., 2010) (Figure 1A), which was evaluated by a global aggregation analysis under chaperone-free conditions using a reconstituted *E. coli* cell-free translation system (PURE system) (Niwa et al., 2009).

#### 3.4.2 Molecular weights and amino acid preferences

As with other features, the Class IV clients, which are limited to those with molecular weights less than approximately 70 kD and can fit within the chaperonin cavity, exhibit a weak yet significant enrichment in alanine/glycine residues. On average, the Ala/Gly enrichment corresponded to six additional alanine or glycine residues in a 300 amino acid protein (Fujiwara et al., 2010).

#### 3.4.3 Structural preferences

A bioinformatic analysis revealed specific structural tendencies among the Class IV clients, with nearly half (25/57) of them displaying the TIM-barrel fold (c.1 in SCOP database terminology), which has been proposed as the preferred folding topology for GroE interactors (Houry et al., 1999; Kerner et al., 2005). Other fold classes, such as the FAD/NAD(P)-binding domain (c.3), the PLP-dependent transferase-like fold (c.67), and the thiolase fold (c.95), were also overrepresented in Class IV, but to a lesser extent than the TIM-barrel fold proteins. Strikingly, all of the fold classes overrepresented among the Class IV members are aggregation-prone folds, as characterized in the global aggregation assay under chaperone-free conditions (Niwa et al., 2009).

#### 3.4.4 Functional preferences

Approximately 70% of Class IV clients are metabolic enzymes, with six of them (DapA, ASD, MetK, FtsE, HemB, and KdsA) being essential for the viability of *E. coli* (Fujiwara et al., 2010). If these are the only essential clients of GroE, creating an *E. coli* strain that is not dependent on GroE should be possible by complementing these six genes; *e.g.,* the conversion of GroE-dependency (Masters et al., 2009; Fujiwara et al., 2010). A viable *groE*-knockout *E. coli* strain would provide an answer to the question of why GroE is essential for cell viability.

## 4 Determinants that define the chaperonin GroEL dependency

After the *in vivo* obligate GroE clients have been identified, the next challenge is to distinguish the determinants that define such GroE dependence. Although these determinants are not yet fully understood, some attempts are introduced here.

### 4.1 Factors associated with GroE dependency

Several attempts have been made to distinguish GroE clients from other proteins, by bioinformatics and experimental approaches (Masters et al., 2009; Tartaglia et al., 2010; Azia et al., 2012). The identification of *in vivo* obligate GroE clients raises the question about the key factors that define the GroE dependency. So far, various approaches have been used to extract the characteristics of the GroE clients. Although they were not sufficient to enable the development of a highly accurate predictor, the following are some factors that are associated with GroE clients. At the amino acid sequence level, bioinformatic approaches were conducted to identify “binding motifs” of GroE clients using sequence patterns similar to the GroES mobile loop segment that binds GroEL (Chaudhuri and Gupta, 2005; Stan et al., 2005). Many other indicators have been proposed as features of GroE clients. The studies that used GFP as an artificial GroE client revealed that highly frustrated regions, wherein not all interactions in the native state are optimized energetically, and increased contact order in the client are associated with greater GroE dependence (Bandyopadhyay et al., 2017, 2019). Also, we note that the features that identify the GroE dependency of a given protein do not necessarily translate broadly to *in vivo* clients. For example, contact order did not distinguish *in vivo* clients from other *E. coli* proteins (Noivirt-Brik et al., 2007).

### 4.2 Converting the GroE dependency of a protein

One way to explore the factors that determine GroE dependence is to convert a protein that is not a GroE client into a GroE client. The Class IV orthologs (MetK, DeoA and YcfH) in the GroE-lacking organism *Ureaplasma urealyticum* fold into the native state in GroE-depleted *E. coli* cells (Fujiwara et al., 2010; Georgescauld et al., 2014). Among them, the MetK ortholog in *Ureaplasma urealyticum* (*Uu*MetK), which shares 45% identity with *E. coli* MetK (*Ec*MetK), has been investigated to decode the determinants for the GroE requirement (Ishimoto et al., 2014). *Uu*MetK does not require GroE during the folding process in *E. coli* (Fujiwara et al., 2010; Fujiwara and Taguchi, 2012). Analyses of chimeric or randomly mutagenized *Uu*MetK genes expressed in GroE-depleted *E. coli* revealed that multiple independent point mutations or even single mutations were sufficient to change from the GroE-independent *Uu*MetK to the GroE-dependent *Uu*MetK, suggesting that subtle differences determine the GroE-dependency. The locations of the mutations in *Uu*MetK were spread out throughout the open reading frame. Notably, the GroE-dependency was well correlated with the tendency of the mutant proteins to form protein aggregates during folding (Ishimoto et al., 2014). Combined with the recent finding that point mutations can convert an aggregation-prone GroE client into a GroE-independent folder (Taguchi et al. unpublished), the differences between GroE clients and non-GroE clients would be subtle, suggesting that they could be interconvertible (Figure 1B).

## 5 Implications in protein evolution: GroE might be required for “newcomer” proteins

The identification of the obligate GroE clients revealed that more than 70% (42 out of 57) are likely to be involved in metabolic reactions. What is the relationship between GroE and metabolic enzymes? A bioinformatics analysis of the metabolic pathways revealed that, as the GroE dependency increases, the clients are more laterally distributed in the metabolic network (Takemoto et al., 2011). In addition, a comparative genome analysis showed that the degree of conservation of GroE clients decreases with the GroE dependence, and the Class I GroE clients are most conserved as compared to other GroE client classes (Takemoto et al., 2011).

These findings could be discussed in the context of protein evolution. Most protein mutations have a negative effect since the protein stability is, in general, marginal (Tokuriki et al., 2008). It has been proposed that chaperones play a role in facilitating protein evolution by buffering the destabilizing mutations that cause misfolding (Rutherford and Lindquist, 1998). This concept, which was originally developed from studies on Hsp90 (Rutherford and Lindquist, 1998), has been extended to GroE (Fares et al., 2002; Tokuriki and Tawfik, 2009; Bogumil and Dagan, 2012). Directed evolution experiments revealed that GroE overexpression could buffer the destabilizing mutations, thereby promoting the folding of compromised proteins to improve their enzyme activities (Tokuriki and Tawfik, 2009; Wyganowski et al., 2013). Indeed, the evolved enzymes in GroE-overexpressing *E. coli* gained aggregation-prone properties and were converted to GroE-dependent proteins (Tokuriki and Tawfik, 2009), consistent with the MetK case described above (Ishimoto et al., 2014). Therefore, the role of GroE in buffering the aggregation-prone mutations helps the destabilized proteins to function in the cellular environment. The buffering effect would allow the destabilized proteins to exhibit novel cellular functions in a chaperone-dependent manner, eventually contributing to the molecular evolution of the protein (Takemoto et al., 2011).

The possible evolutional role of GroE prompted us to investigate whether GroE clients are conserved among species. If the obligate GroE clients (Class IV) have evolutionarily acquired some function or other traits more recently by mutations of existing proteins than other proteome members, then we would expect that the GroE clients are not conserved. So far, an extensive survey to identify *in vivo* obligate GroE-dependent clients has only been conducted in *E. coli.* For verification, we await a study to identify the *in vivo* clients in other bacteria, at the level of that in *E. coli.*

## 6 Future perspectives

Even though there have been great breakthroughs in highly accurate protein structure prediction, as represented by AlphaFold2 (Jumper et al., 2021) and rational *de novo* protein design (Huang et al., 2016), we still do not fully understand the protein folding process. One of the obstacles is protein aggregation. Protein aggregation is a notorious problem when handling proteins and has often been ignored as an unwanted side reaction in protein folding. The chaperone studies unveiled the previously unrecognized role of aggregation-prone proteins in the protein world. Since Anfinsen demonstrated the basic principle of protein folding (Anfinsen, 1973), extensive efforts to elucidate the protein folding mechanism have been performed for more than half a century (Dill and MacCallum, 2012). However, the “folding” mechanisms of “recalcitrant” proteins, which are not spontaneously folded under chaperone-free conditions, remain enigmatic. Most physicochemical methods such as stopped-flow kinetic experiments and calorimetric measurements are not amenable to aggregated proteins. Approaches to tackle the “folding” mechanisms of recalcitrant proteins are necessary to understand the wide spectrum of protein folding in the cell.
